# Development of a rotational set‐up correction device for stereotactic head radiation therapy: A performance evaluation

**DOI:** 10.1002/acm2.12616

**Published:** 2019-05-21

**Authors:** Keisuke Usui, Akira Isobe, Naoya Hara, Tomoya Muroi, Osamu Sajiki, Koichi Ogawa, Naoto Shikama, Keisuke Sasai

**Affiliations:** ^1^ Department of Radiation Oncology Juntendo University Tokyo Japan; ^2^ Department of Radiology Juntendo University Hospital Tokyo Japan; ^3^ Engineering System Co., Ltd Nagano Japan; ^4^ Faculty of Science and Engineering Hosei University Tokyo Japan

**Keywords:** head supporting device, helical tomotherapy, multiple targets, stereotactic radiotherapy

## Abstract

We developed a new head supporting device to provide accurate correction of rotational setup during image‐guided radiation therapy (IGRT), evaluating its correction performance and the efficacy of dose distribution in stereotactic radiotherapy (SRT) using a helical tomotherapy (HT) system. The accuracy of rotational motion was measured using an electronic inclinometer; we compared device angles and measurement values from 0.0° to 3.0°. The correction accuracy was investigated based on the distance between rotational centers in the device and on megavoltage computed tomography (MVCT); the correction values were compared using distances in the range of 0.0–9.0 cm using a head phantom with a rotational error of 1.5°. For an SRT with a simultaneous integrated boost plan and a rotational error of 3.0° in yaw angle using a head phantom, and for a single‐isocenter SRT for multiple brain metastases in the data of three patients, dosimetric efficacy of the HT unit was evaluated for calculated dose distributions with MVCT after rotational correction. This device can correct pitch and yaw angles within 0.3° and can be corrected to within 0.5° for each rotational angle according to the result of MVCT correction regardless of the rotational center position. In the head phantom study, the device had a beneficial impact on rotational correction; D99% for the target improved by approximately 10% with rotational correction. Using patient data with the device, the mean difference based on the treatment planning data was 0.3% for D99% and −0.1% for coverage index to the target. Our rotational setup correction device has high efficacy, and can be used for IGRT.

## 1 INTRODUCTION

Stereotactic radiation therapy (SRT) can be used to treat several benign and malignant diseases using highly conformal dose distributions.^1^ The volumetric modulated arc therapy technique allows simultaneous treatment of multiple targets using a single plan with one isocenter.^2^, ^3^, ^4^, ^5^

Helical tomotherapy (HT) (Tomo HD system®, Accuray, Sunnyvale CA, USA) involves a beam delivery system capable of achieving highly conformal dose distributions with good coverage of targets and normal tissue sparing. Several studies have reported on the use of HT in the treatment of multiple brain metastases by using SRT with the simultaneous integrated boost (SIB) technique. In SRT, increases in rotational setup error in patient positioning and changes in the distance of the target to the rotational center may be associated with significant dosimetric uncertainties in multi‐target, single‐isocenter SRT treatments.^3^, ^6^ This necessitates patient setup position to be corrected in six directions using an in‐room imaging system in conjunction with a linac‐based treatment couch.^7^ However, rotational setup errors in pitch and yaw angles cannot be corrected in image‐guided radiation therapy (IGRT) using current HT systems. Although rotational error information can be acquired using pre‐treatment megavoltage cone beam computed tomography (MVCT), patient rotational errors cannot be modified on the fly.

In this study, we developed a new head support device that allows for accurate rotational setup correction of pitch and yaw angles for IGRT for head SRT using the HT system. To evaluate the accuracy of rotational correction, we investigated the operation and correction accuracies depending on the distance between the centers of rotation of the device and the MVCT image. We assessed the dosimetric efficacy of rotational correction for head SRT using the HT unit with both a head phantom and human data.

## 2 MATERIALS AND METHODS

**Figure 1:**
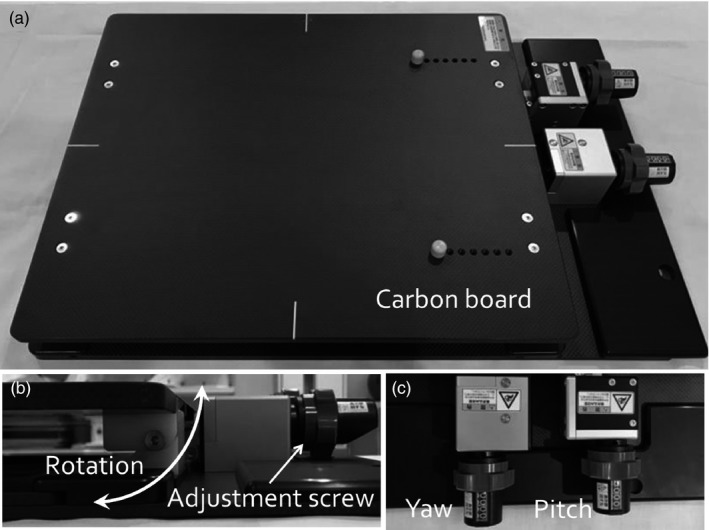
A rotation correction device for head stereotactic radiotherapy. This device can correct for rotational error in pitch and yaw angles using two screws placed every 0.1°.

**Figure 2:**
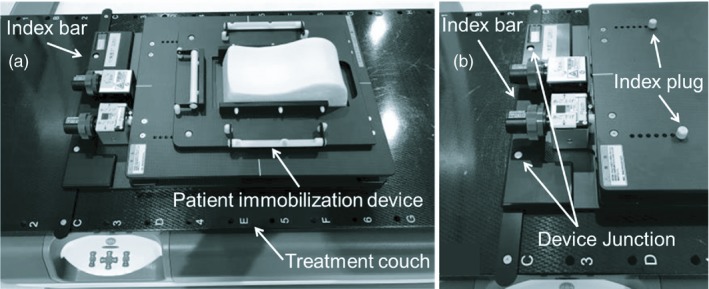
(a) Connecting the patient immobilization device to the treatment couch. This device is fitted onto the couch by modifying the index bar position connected with the device junction. The patient immobilization device is connected by the index plug, and can be modified by changing the index plug’s position [Fig. 2(b)].

The head supporting device is shown in Fig. 1(a). This device is constructed from carbon material, and can be rotated in 0.1° increments for pitch and yaw angles using two screws [Figs. 1(b) and 1(c)]. By placing the commonly used head shell system on the top of this device, we can rotate the head position during head fixation. Using an index bar, the device is fitted onto the treatment couch in the specific position shown in Fig. 2(a). The patient immobilization device is connected by an index plug shown in Fig. 2(b). Moving the position of the index plug allows adjustment of head shell position. This device may be used during CT data acquisition for treatment planning as well as during beam irradiation. The device’s contour will be contained in the planning CT image data, allowing modeling of beam attenuation and changes in surface dose in any treatment planning system for dose calculation. This device passed tests of mechanical rotational motion, load carrying, and deflection at the time of construction. Therefore, we have confirmed that this device has a safety function to be used.

### 2.1 Commissioning of the rotation correcting head supporting device

#### 2.1.1 Rotational motion accuracy

**Figure 3:**
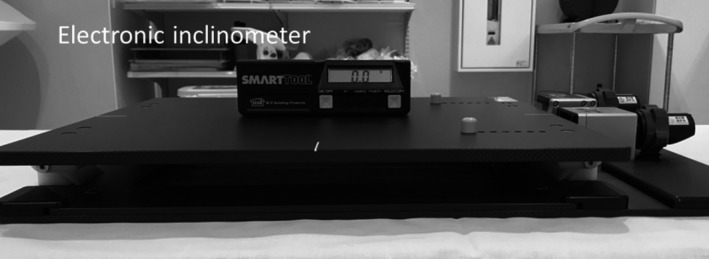
Experimental setup for rotational motion accuracy measurement in pitch and yaw angles.

The rotational motion accuracy of this device was validated using an electronic inclinometer (SmartTool Builder’s Angle Sensor Module®, M‐D Building Products, Inc., Mississauga, Canada) along two independent axes (pitch and yaw angles). Figure 3 shows the experimental setting, where we compared device angles and measurement values in the range of 0.0°–3.0°.

#### 2.1.2 Consistency test with MVCT correction value

**Figure 4:**
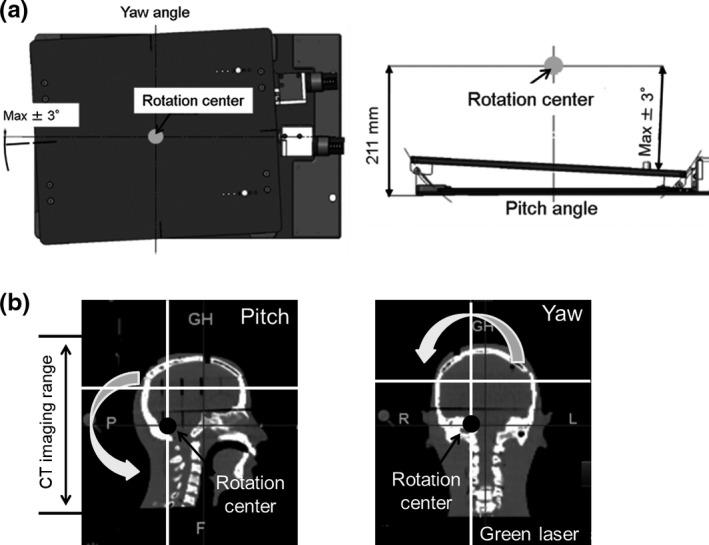
Rotational center position of the device (a) and the megavoltage computed tomography (MVCT) (b). In the device, the rotational center is fixed, and put in the center of the rotating plate in the translation direction, and 211 mm above the bottom of this device in the vertical direction. In MVCT with the helical tomography unit, rotational position is determined on the position of the green laser (bold lines) in roll and pitch angles, and the center of CT image acquisition in the pitch angle.

To validate the consistency of rotational correction in this device, we used the MVCT registration result. We used a human head phantom (RAN110®, Alderson Research Laboratories, Long Island City, NY, USA). Rotational setup errors of 0.0°–3.0° in the pitch and yaw angles were added to the device’s position. MVCT image data from the HT unit were acquired in this manner, and differences from the reference image were measured, allowing the comparison of the preliminary error value with the measured correction values in each rotational direction. In the MVCT image registration process, an auto‐registration algorithm was applied for bone structure matching. In this study, consistent rotational centers of the device and MVCT were maintained. Figure 4 shows the centers’ position of rotation in the device (a) and the MVCT (b). The rotational center of the device is located at the center of the rotating plate in the transverse direction, and 211 mm above the bottom edge of this device in the vertical direction. The rotational center for image registration using the MVCT is dependent on the treatment plan in the HT system, with rotational centers of directions in roll and yaw indicated by the position of a green laser marker. Pitch direction is indicated in the center of the CT image acquisition range [Fig. 4(b)]. To maintain consistency of rotation centers in the pitch and yaw angles, we adjusted the position of the laser and the center of CT data acquisition range with the device center.

### 2.2 Correction accuracy based on the distance between rotational centers

The correction accuracy on the basis of the distance between rotational centers of the device and MVCT acquired using the HT unit was evaluated in the direction of the pitch and yaw angles. The rotational correction error value is dependent on the distance between the rotational center of device and the position of the rotational center of the MVCT, therefore, we evaluated correction accuracy by changing the distance between each rotational center in the range of 0.0–9.0 cm by modifying the position of the rotational center of the MVCT in the treatment plan.

**Figure 5:**
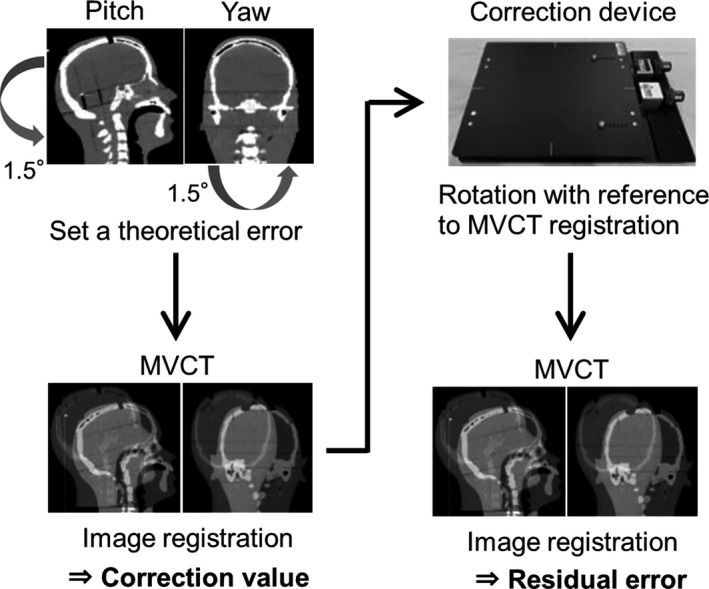
Workflow of the correction accuracy on the basis of the distance between rotational centers. Correction values acquired from megavoltage computed tomography (MVCT) were compared with ideal error (1.5°), and residual error was measured with repeat image‐guided radiation therapy following rotational correction.

Figure 5 shows the workflow for quantifying the correction accuracy using the device. The human head phantom is positioned with theoretical setup error values of 1.5° in the pitch and yaw directions using the device’s rotation screw. We measured the rotation value from the MVCT image registration in six dimensions compared with planning computed tomography (CT) images without rotational errors. This correction value was compared with the theoretical setup error value of 1.5°. After correction of the rotational error by the device, we then measured the amount of residual correction error values in the IGRT process again.

### 2.3 Using a rotation‐correcting head support device for head SRT

#### 2.3.1 Head phantom study

**Figure 6:**
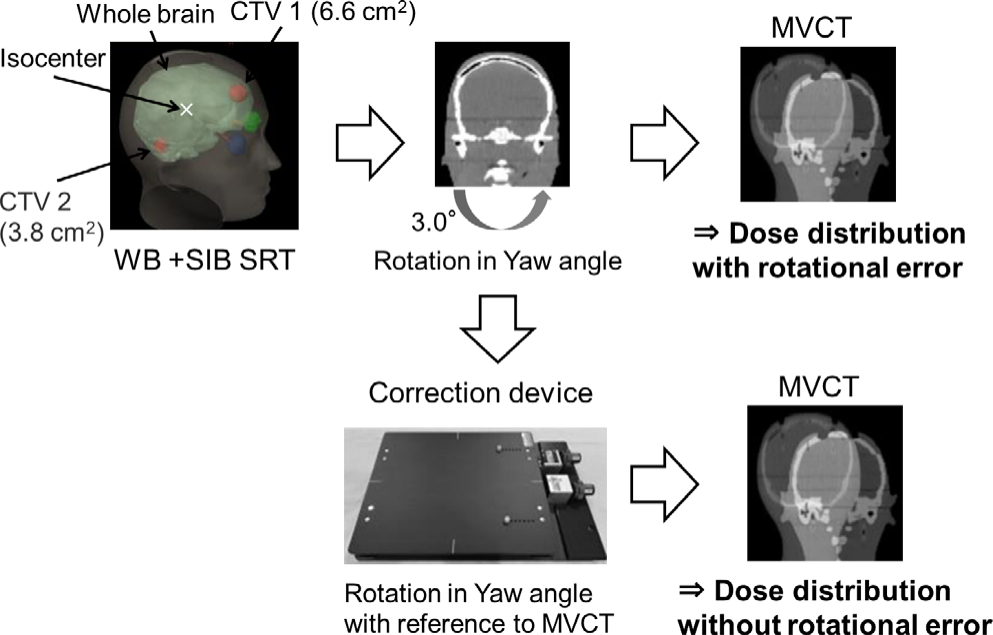
Dosimetric efficacy with rotational correction using the device by calculated dose distributions in megavoltage computed tomography (MVCT). Dose distributions with and without correction in MVCT were compared.

An SRT plan with SIB was created with 40 Gy in four fractions prescribed for D99% to two clinical target volumes (CTVs) and 20 Gy in four fractions for D95% to the whole brain using the planning station (TomoHD System ver. 2.1.0®, Accuray, Sunnyvale CA, USA); the calculation grid size was 2.0 × 2.0 mm^2^, the field width was 2.0 cm, the rotation pitch was 0.287 and the modulation factor was 2.5. The volume of the two CTVs were 6.6 cm^2^ for CTV 1 and 3.8 cm^2^ for CTV 2. In this study, the rotational center of the device and the MVCT image being identical represented the ideal situation. Dosimetric efficacy with rotational correction was evaluated by dose volume histogram (DVH) analysis using a calculated dose distribution with MVCT after rotational correction using the device. For dose calculation, a conversion curve for MVCT values to relative electron density value was generated using an electron density phantom (RMI‐467®; GAMMEX RMI GmbH, Biebertal, Germany). Figure 6 shows the workflow of dosimetric efficacy investigation. The head phantom was located with a theoretical set‐up error value of 3.0° in the yaw direction. Rotational error was corrected using our device referenced to the MVCT registration results. Dose distributions with and without correction in MVCT were compared.

#### 2.3.2 Analysis of patient data

We retrospectively analyzed data from three patients treated by single‐isocenter SRT for multiple brain metastases using the HT unit. The SRT plans were created with 30 Gy in three fractions prescribed for D99% to the CTVs using the planning station; with the same conditions of dose calculation as those in Section 22.6.C.12.6. The number of brain metastases was two in each case, and the volume of CTVs and PTVs were 0.1–9.5, and 0.7–25.8 cm^3^, respectively. In each treatment, the required rotational setup correction values were determined using the MVCT images before irradiation. Residual correction values were quantified after rotational correction by the device. Dosimetric efficacy was evaluated using dose parameters using D99% and coverage index (CoI) to the CTVs as indicated by the dose distributions in the MVCT after rotational correction. The CoI was calculated based on the volume of the targets (CTV and PTV) and the volume receiving the prescribed dose of 30 Gy. Dose distribution was compared between four‐dimensional (4D) (three horizontal axes and roll angle) and six‐dimensional (6D) (three horizontal axes and pitch, roll, and yaw angles) correction. Structures of the CTV and PTV were contoured in the MVCT image, which were aligned with the CT image based on the bone positions.

## 3 RESULTS

### 3.1 Performance of rotational motion

#### 3.1.1 Rotational motion accuracy

Table 1 shows the results of rotational motion accuracy in the head supporting device. Rotational motion accuracy was indicated by the difference in values of the device angle and measurement values of the electronic inclinometer. This measurement was repeated five times, and the mean value and standard deviation (SD) were obtained.

**Table 1:** Rotational motion accuracy of the device. We obtained deviation values between the device angle and the electronic inclinometer value. Deviation value was calculated by subtracting the electronic inclinometer value from the device angle. This measurement was repeated five times and the mean deviation calculated.

Device angle (°)	Pitch (°)		Yaw (°)	
	Mean deviation	SD	Mean deviation	SD
0.5	0.1	0.1	−0.1	0.0
1.0	0.0	0.1	0.1	0.1
1.5	0.0	0.2	−0.2	0.0
2.0	0.0	0.1	0.0	0.1
2.5	0.0	0.1	−0.1	0.0
3.0	0.0	0.1	−0.1	0.0
3.5	0.1	0.0	−0.1	0.0
−0.5	0.0	0.1	−0.1	0.1
−1.0	0.1	0.3	0.1	0.1
−1.5	0.0	0.2	0.0	0.3
−2.0	−0.1	0.2	−0.1	0.1
−2.5	−0.2	0.1	−0.1	0.0
−3.0	−0.3	0.1	−0.2	0.1
Mean	0.0		−0.1	
SD	0.1		0.1	

#### 3.1.2 Consistency test with MVCT correction values

Table 2 shows results of the consistency test for the device. Rotational angles measured by the device were in agreement with MVCT image correction values ±0.2°.

**Table 2:** Consistency between rotational angle in the device and the results of megavoltage computed tomography (MVCT) image registration. We obtained deviation values calculated by subtracting the MVCT registration value from the ideal rotational value of the device.

Ideal rotation error (°)		Deviation (°)	
		Pitch	Yaw
Pitch	0.1	0.0	0.1
	0.3	−0.1	0.0
	0.5	0.0	0.2
	0.8	0.0	0.0
	1.0	−0.1	0.0
	1.3	0.0	0.1
	1.5	−0.1	0.0
	1.7	0.0	0.0
	2.0	−0.2	0.2
	3.0	0.0	0.0
Yaw	0.1	0.0	0.2
	0.3	0.0	0.2
	0.5	0.0	0.2
	0.8	−0.1	0.0
	1.0	0.0	0.0
	1.3	−0.1	0.0
	1.5	0.1	−0.2
	1.7	−0.1	0.2
	2.0	−0.1	0.1
	3.0	0.0	0.0

### 3.2 Correction accuracy in the process of IGRT

Table 3 shows the results of correction accuracy based on the distance between rotational centers. The initial rotational angle of the device and rotational correction value agreed within 0.5°, and the residual error after rotation correction was also within 0.5°.

**Table 3:** Correction accuracy depending on each difference of rotational center position. Correction value was compared with theoretical rotation angle of the device and results of megavoltage computed tomography (MVCT) image registration. Residual error was obtained with the MVCT registration value after rotational correction using the device.

Device center to MVCT center (cm)	Deviation (°)			
	Correction value		Residual error	
	Pitch	Yaw	Pitch	Yaw
Vertical				
0.0	0.1	−0.2	0.5	0.3
3.0	0.0	−0.2	0.0	0.0
6.0	0.2	0.0	0.0	0.2
9.0	0.0	0.0	0.0	0.2
Lateral				
0.0	0.1	0.2	0.5	0.3
1.0	−0.3	0.3	0.1	−0.4
2.0	0.3	0.1	0.5	−0.5
Longitudinal				
0.0	0.1	−0.2	0.0	0.3
1.0	−0.3	0.3	−0.1	0.3
2.0	−0.3	0.0	0.0	0.0
3.0	0.1	−0.1	−0.1	0.0
Mean of absolute value	0.2	0.1	0.2	0.2
SD	0.2	0.2	0.2	0.3

### 3.3 Setup position and dosimetric efficacy of utilizing the device for head SRT

#### 3.3.1 Head phantom study

**Figure 7:**
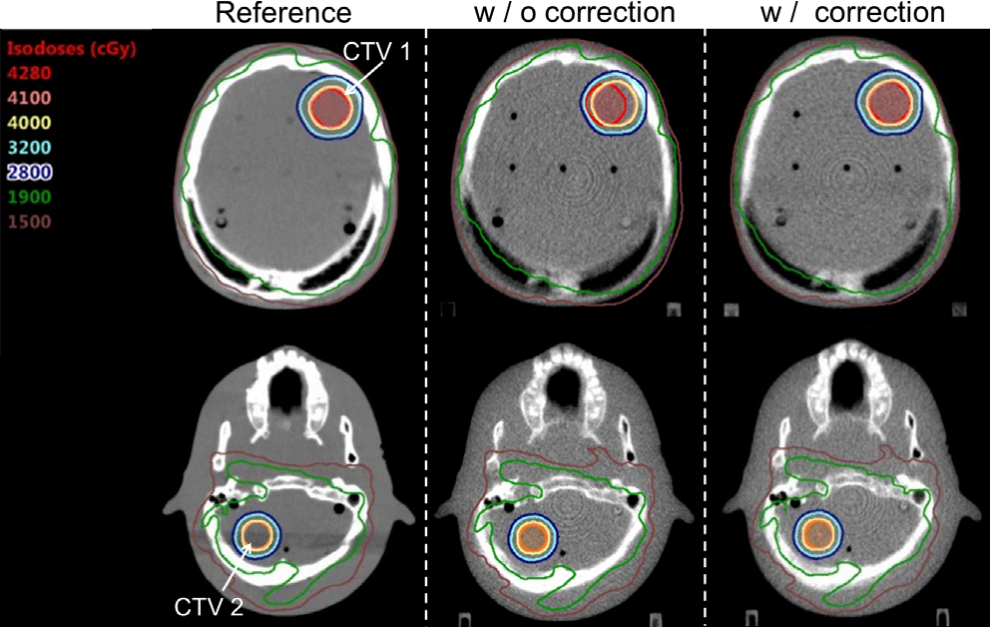
Dose distributions in whole brain + simultaneous integrated boost (SIB) stereotactic radiotherapy calculated on multi‐slice computed tomography (reference) and megavoltage computed tomography (with/without rotational correction).

**Figure 8:**
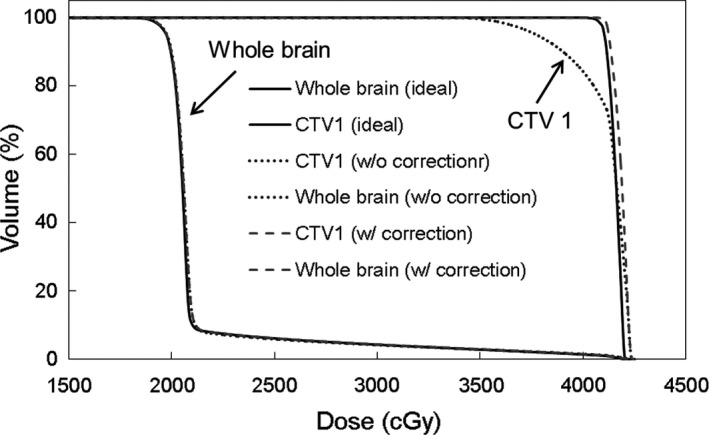
Results of the dose volume histogram analysis. Dose distribution for whole brain was similar to that of the ideal multi‐slice computed tomography plan. Conversely, the dose distribution for clinical target volume (CTV) 1 degraded with rotational error; the D99% for CTV 1 improved by approximately 10% using our device.

Figures 7 and 8 shows the dose distribution and the DVH curve in whole brain + SRT SIB calculated on multislice CT as a reference, and on the MVCT with and without rotational correction. Dose distribution for the whole brain and CTV 2 were similar to those of the ideal CT plan. Conversely, the dose distribution for CTV 1 degraded with rotational error; however, the D99% for CTV 1 improved by approximately 10% using developed device.

#### 3.3.2 Real human data

Tables 4 and 5 show the rotational correction values prior to and after treatment beam irradiation. The maximum correction value in pitch and yaw was 2.9°. Table 6 shows the dose parameters with 4D and 6D setup correction. In this table, difference rates for each dose parameter were calculated based on treatment planning data, and averaged over each CTV and PTV region. The results of CoI to CTV showed significantly smaller differences from the treatment planning data. The mean difference rate in 4D correction was −1.0% and −2.4% for D99% and CoI to CTV. The rates in 6D correction were 0.3% and −0.1%, respectively.

**Table 4:** Rotational correction values prior to treatment beam irradiation.

Rotational direction	Pitch	Roll	Yaw
Mean	1.2	0.9	1.3
Standard deviation	1.7	1.2	1.6

**Table 5:** Rotational correction values following treatment beam irradiation.

Rotational direction	Pitch	Roll	Yaw
Mean	0.3	0.2	0.3
Standard deviation	0.4	0.4	0.3

**Table 6:** Difference rate of dose parameters from treatment planning data in four‐dimensional (4D) and six‐dimensional (6D) setup correction. This result becomes very small with decreasing difference from the treatment planning.

Target	Correction	Difference rate from treatment planning [%]	
		D99	CoI
Patient #1			
CTV	4D	−1.7	−3.1
	6D	−0.5	0.0
PTV	4D	−10.5	−19.2
	6D	−6.7	−12.2
Patient #2			
CTV	4D	−1.1	−3.0
	6D	1.1	−0.2
PTV	4D	−5.8	−1.7
	6D	−3.6	−0.5
Patient #3			
CTV	4D	0.4	0.0
	6D	0.6	0.0
PTV	4D	0.2	0.1
	6D	0.7	0.5

## 4 DISCUSSION

In this study, we assessed a new rotational setup correction device and investigated the usability of this device referring to results of MVCT image registration in the process of IGRT using the HT system. As shown in Table 1, measurements of rotational motion matched the electronic inclinometer results within ±0.3°. Therefore, our results are sufficient for use of patient setup correction in head SRT, because rotations of 0.5° exerted only a minimal effect on target coverage.^3^, ^7^, ^8^

As shown in Tables 2 and 3, rotation values were consistent with these values measured by the MVCT image with an accuracy of ±0.5°. In some cases, the residual error values were greater than the deviations of correction values; these large residual errors were caused by differences between the accuracy of the MVCT image registration algorithm and the rotation accuracy of our device. However, the device can be used for correction in reference to the results of MVCT registration with an accuracy of ±0.5° regardless of each rotational center position, because positional correction values calculated by the MVCT are modified in rotational and translational movement using the treatment couch in the HT unit. Recently, several six‐degrees‐of‐freedom (6‐DoF) couch systems capable of correcting for three orthogonal rotations (pitch, yaw, and roll angles) have been employed.^9^, ^10^ Combining this device with 6‐DoF couch systems will allow assessment of the different degrees of rotation of articulated parts of the body, such as the head and the neck, separately. The developed device can be used with any treatment modality and in conjunction with any radiation treatment device without complex positioning procedures.

To quantify how much target coverage can be affected in a worst‐case situation, the 3.0° of rotational error in yaw angle was selected based on prior studies of rotational setup errors. As shown in Fig. 7, rotations of 3.0° were found to have enormous impact on target coverage. In patient data analysis, the rotational correction value before irradiation for pitch was 1.2° ± 1.7°, for roll: 0.9° ± 1.2°, and for yaw: 1.3° ± 1.6°. Therefore, rotational setup correction was needed for head SRT using the HT unit. Dose parameters were improved by 6D setup correction using the device; therefore, the mean difference in CoI in target dose was improved by 6D setup correction. In the results of patient #3, no significant improvement using the device was observed, because the CTV was large (9.5 cm^3^). In a previous study, in a case with small target volume, rotational setup error critically affected the target doses.^6^ To irradiate small target regions, our device had a beneficial impact on the rotational setup correction and irradiated dose distribution for head SRT using the HT system. Boswell et al. showed a novel rotational correction method in the HT unit by using very slow continuous couch motion in a direction perpendicular to the scanning direction.^11^ However, this is not implemented in current HT systems. In situations where the new 6‐DoF couch system is integrated into the HT system, its rotational accuracy, correctable rotation angle, and range are unclear.

The mean transmission factor of our developed device is about 1.0% with 1 arc irradiation with a 6 MV beam. This slight attenuation can be adjusted for in the treatment planning by including the contour of this device in the planning CT data. Patients can move in their head mask during treatment.^12^ As shown in Table 5, patient movement during the treatment was speculated small motion. However, this device can improve the accuracy of these inter‐fractional motions without extending patient setup time, although it cannot monitor patient motion during irradiation. For accurate radiation treatment using SRT for the head region, intra‐fractional motion also has to be controlled.^13^ Therefore, repeat intra‐fractional imaging and patient set‐up correction, and real‐time monitoring devices, have to be combined with our device for reduction in the errors associated with intra‐fractional patient movement.

## 5 CONCLUSION

We developed a new rotational set‐up correctable device for head SRT with an operational accuracy within 0.3°. This device had a beneficial impact on the rotational setup correction and irradiated dose distribution for head SRT, and can be corrected to within 0.5° for pitch and yaw angle errors according to the degree of IGRT correction.

## CONFLICT OF INTEREST

No conflict of interest.
